# Balancing between cuproplasia and copper-dependent cell death: molecular basis and clinical implications of ATOX1 in cancer

**DOI:** 10.1186/s13046-025-03486-5

**Published:** 2025-07-28

**Authors:** Justyna Suwara, Mariusz L. Hartman

**Affiliations:** Department of Molecular Biology of Cancer, Medical University of Lodz, 6/8 Mazowiecka Street, Lodz, 92-215 Poland

**Keywords:** ATOX1, Cancer, Copper, Copper chaperone, Copper homeostasis, Cuproplasia, Cuproptosis, Oxidative stress

## Abstract

Human antioxidant protein 1 (ATOX1) is an essential regulator of copper homeostasis in cells. By interacting with other proteins involved in controlling the intracellular levels of cuprous ions (Cu^+^), ATOX1 contributes to the import, export, and subcellular distribution of Cu^+^ as it functions within the CTR1-ATOX1-ATP7A/ATP7B axis. For this reason, ATOX1 plays a key role in preventing copper toxicity. Since copper ions have been shown to regulate the activity of a subset of other signaling proteins, ATOX1 can support cell proliferation, migration, and survival. Notably, ATOX1 is the only identified copper chaperone that has transcription factor activity. In this respect, *CCND1*,* MDC1*,* NCF1*,* PPA2*, and *SOD3* have been experimentally validated as transcriptional targets of ATOX1 in distinct types of cells. The multifaceted actions of ATOX1 indicate that its dysregulation can lead to changes in the activity of crucial signaling pathways associated with diverse disorders, including cancer. Indeed, ATOX1 levels are frequently increased in cancer as demonstrated in multiple studies and supported by data available in GEPIA. ATOX1 has been implicated in cancer biology because of its role in the proliferation and metastatic spread of cancer cells and protection from oxidative stress. Additionally, ATOX1 may impact the drug response and resistance of cancer cells by influencing detoxification mechanisms as demonstrated for platinum-based therapies. In turn, the role of ATOX1 in the susceptibility of cancer cells to targeted therapies and immunotherapy remains elusive. This, however, should be a direction of further research considering the recent advances in understanding the complex role of copper in cancer cells, which can be associated with either protumorigenic effects (cuproplasia) or the induction of novel copper-dependent regulated cell death (cuproptosis) to combat cancer cells. Therefore, the disruption of ATOX1-mediated processes could be beneficial for the efficacy of anticancer therapies, although this possibility should be treated with caution because of the dual role of copper in cancer. Moreover, the prognostic value of *ATOX1* expression for the clinical outcome of cancer patients needs to be clarified. In this review, we summarize the current state of knowledge about ATOX1 in cancer focusing on its molecular aspects and potential clinical implications.

## Background

Copper (Cu) is one of the most prevalent transition metals in living organisms and plays an essential role as a cofactor for many cellular cuproproteins to facilitate molecular transport, catalyze biochemical reactions, and regulate the activity of several signaling molecules [1]. Copper ions are transported into the cells after the reduction of cupric (Cu^2+^) to cuprous (Cu^+^) cations, and must be distributed within the cell in a well-controlled manner as cuprous ions can be toxic because of their ability to generate reactive oxygen species (ROS) through Fenton-like reactions by interacting with hydrogen peroxide to produce particularly reactive hydroxyl radicals (•OH) [2, 3]. For this reason, copper homeostasis is tightly regulated by copper-binding proteins such as ceruloplasmin and transporters that mediate copper uptake, efflux and distribution to copper-dependent proteins and enzymes inside the cell [2]. In brief, copper transporter 1 (CTR1), also known as solute carrier family 31 member 1 (SLC31A1), predominantly mediates the uptake of cuprous ions into the cell, while CTR2 is a low-affinity transporter of Cu^+^ [4, 5]. CTR proteins deliver cuprous ions to distinct metallochaperones, including copper chaperone for superoxide dismutase 1 (CCS1) and nonproteinaceous low-molecular-weight copper ligand (CuL), or copper can be sequestered by glutathione (GSH) and metallothioneins (MTs) [4, 6]. CCS1 contributes to the activation of superoxide dismutase 1 (SOD1) [7, 8], while CuL enters the mitochondria to deliver Cu^+^ for the copper chaperone of cytochrome *c* oxidase 17 (COX17) [6, 9]. In this respect, antioxidant protein 1 (ATOX1, formerly known as HAH1) is a small copper chaperone protein that plays a crucial role in maintaining intracellular copper homeostasis [10–12]. The expression of *ATOX1* can vary across different tissues and developmental stages, whereas ATOX1 is particularly important in tissues that require substantial copper metabolism, including the liver, brain and kidneys [13]. When copper levels in the cell are elevated, *ATOX1* expression is upregulated to ensure a proper supply of copper chaperones to cope with the increased load of copper ions [11]. In this review, we comprehensively discuss the molecular aspects of ATOX1 as well as its key interactions with distinct molecules crucial for cancer development and progression, and highlight the potential clinical relevance of targeting ATOX1 in cancer.

### Overview of ATOX1 structure and function

#### Structure of the ATOX1 gene and protein

**Fig. 1:**
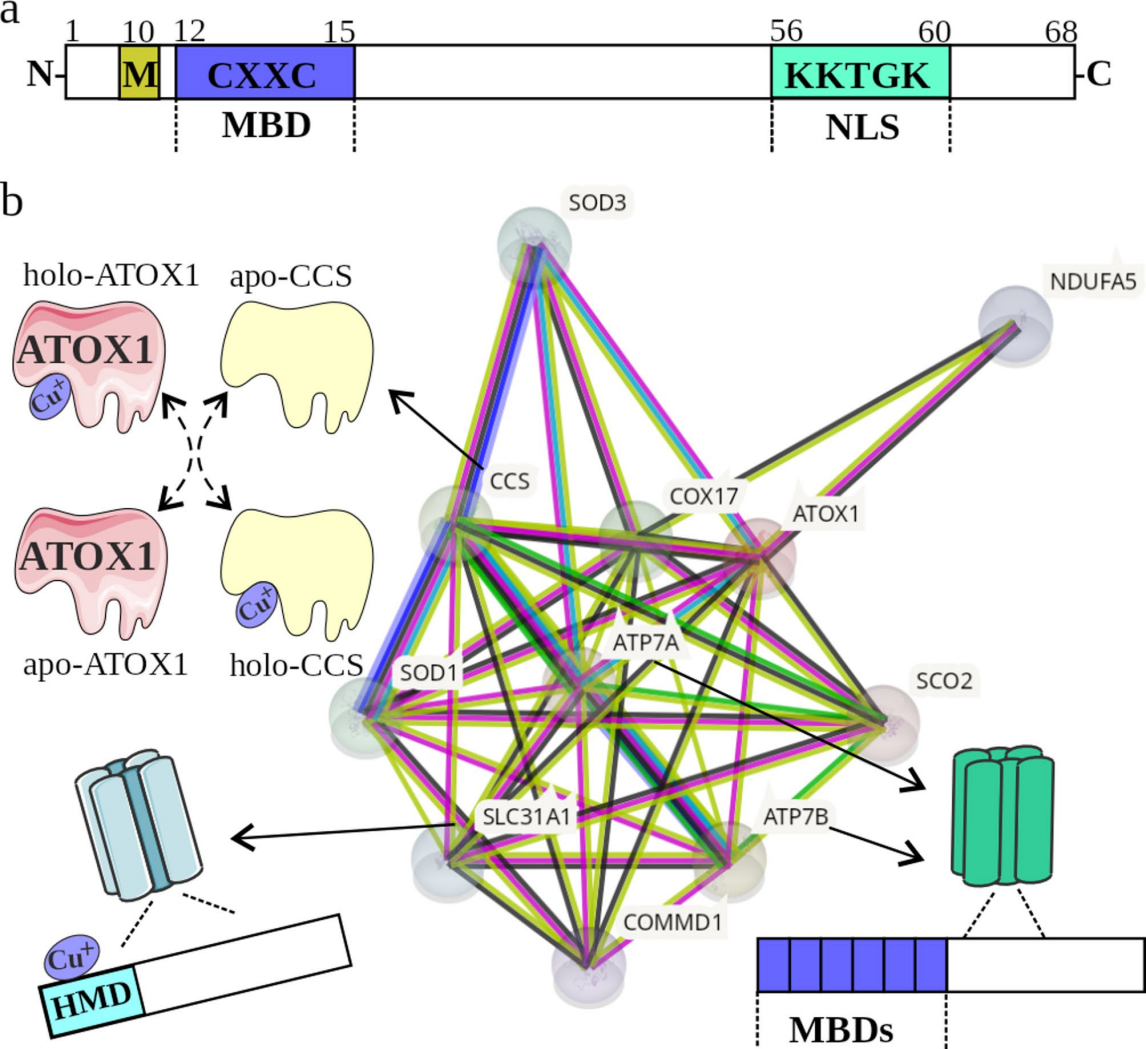
(**a**) Domain structure of ATOX1. The most important functional domains and their amino acid sequences are shown. (**b**) The major binding partners and their interactions with ATOX1 retrieved from STRING Protein-Protein Interaction Networks (https://string-db.org/) and described in the text. ATP7A/ATP7B, copper-transporting ATPase alpha/beta; CCS, copper chaperone for superoxide dismutase; COMMD1, COMM domain-containing protein 1; COX17, cyclooxygenase 17; MBD, metal-binding domain; NDUFA5, NADH: ubiquinone oxidoreductase subunit A5; NLS, nuclear localization sequence; SCO2, synthesis of cytochrome *c* oxidase 2; SLC31A1, solute carrier family 31 member 1; SOD, superoxide dismutase

The characteristics of *ATOX1* and its corresponding protein have increased significantly since its identification in *Saccharomyces cerevisiae* in 1995 and two years later in humans by screening a liver cDNA library and isolating a full-length cDNA encoding ATOX1 [14, 15]. *ATOX1* is located on the long arm of chromosome 5 and consists of three introns and four exons [16]. The structure of the human ATOX1 protein has been extensively investigated via X-ray diffraction and nuclear magnetic resonance spectroscopy, and the structures of ATOX1 loaded with various metal ions, including cadmium, copper, mercury, platinum, silver and zinc have been characterized [17–21]. ATOX1 is a chaperone protein with a weight of 7402 Da and consists of 68 amino acid residues (Fig. 1a) [22, 23]. Structural studies revealed that ATOX1 has a βαβββαβ-ferredoxin-like fold with a surface-exposed Cys-X-X-Cys (CXXC) motif at the N-terminus that binds the metal ion via two cysteine residues: Cys12 and Cys15 [24–26]. The CXXC motif in ATOX1 provides the flexibility necessary to bind and transport cuprous ions to other proteins involved in the utilization or export of copper [27–29]. Owing to various conformational states, ATOX1 switches between the apo-form (unbound to copper) and the holo-form (bound to copper) during its functional cycle, and the properties of both states are crucial for the transport of cuprous ions [26, 27]. It has been shown that Cys15 is an important residue for dimerization of ATOX1, whereas Cys12 is a critical site for the binding of cuprous ions [26]. In addition, the Met10 and Lys60 residues in ATOX1 play essential roles in the retention of cuprous ions in this protein [30], whereas the Lys60 additionally stabilizes the copper-ATOX1 dimer [26]. As evidenced by electron paramagnetic resonance spectroscopy and multiscale simulation studies, the apo-form of ATOX1 can exist in four different conformations, whereas only two holo-forms of ATOX1 have been found [29, 31]. Additionally, ATOX1 contains a lysine-rich region (KKTGK) at its C-terminus with conserved Lys56 and Lys60 residues. This region acts as a nuclear localization sequence (NLS) (Fig. 1a) [32, 33].

**Fig. 2:**
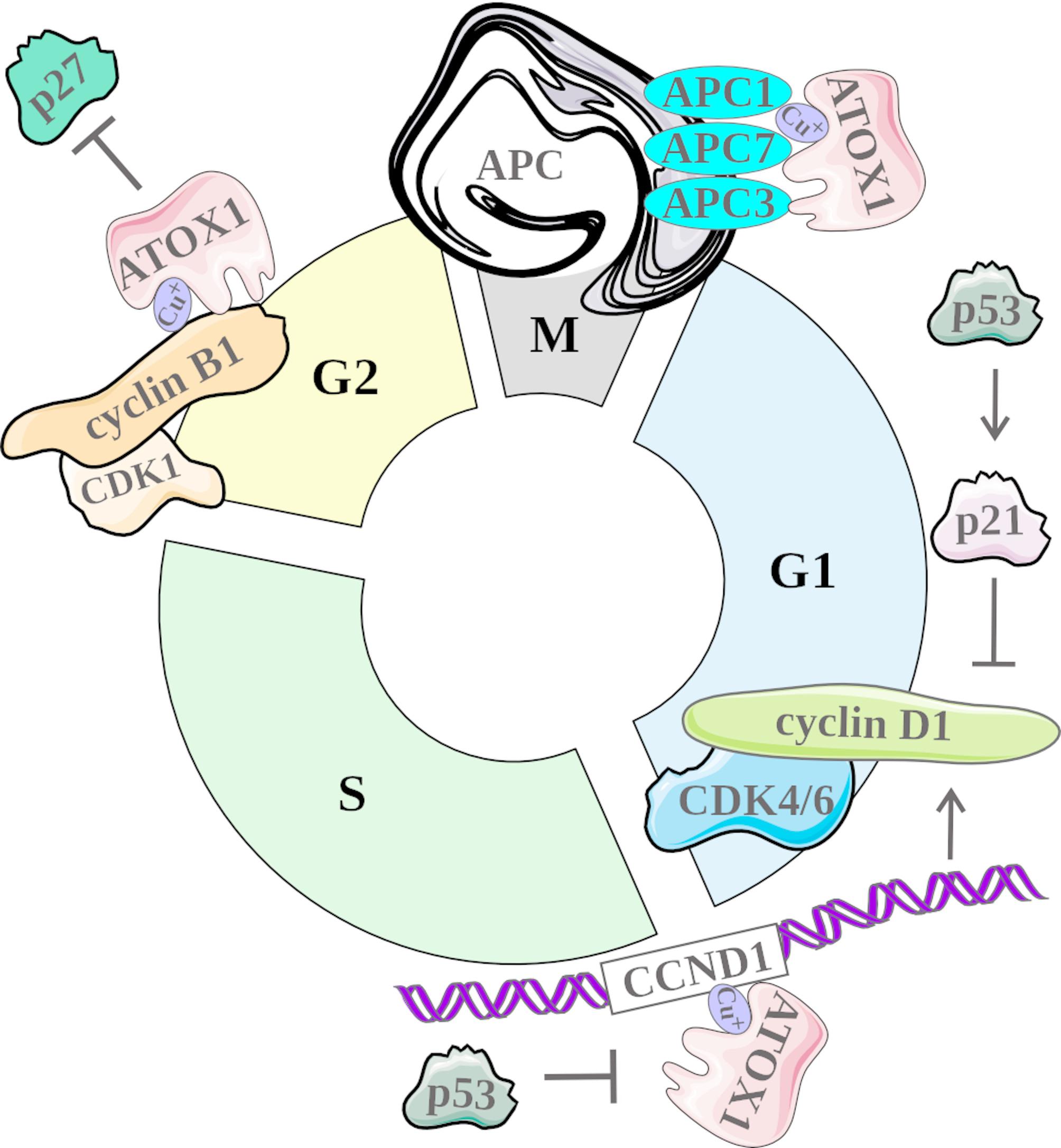
The contribution of ATOX1 to the regulation of cancer cell cycle progression. In the G1 phase of the cell cycle, ATOX can act as a transcription factor for *CCND1* encoding cyclin D1, an important protein for regulating the transition from the G1 phase to the S phase of the cell cycle. p53 can inhibit the transcriptional activity of ATOX1, thereby downregulating *CCDN1* expression. Additionally, p53 can upregulate the level of p21 protein that acts as an inhibitor of cyclin and downstream signaling with CDK4/6. ATOX1 can also be crucial for the transition between the G2 phase and the M phase of the cell cycle by stimulating the expression of the cyclin B1, which can also contribute to the activation of CDK1 by facilitating the transfer of copper ions from ATOX1 to CDK1. ATOX1 can also interact with APC triggering the degradation of proteins that regulate mitotic exit and entry into the G1 phase of the cell cycle. APC, anaphase-promoting complex; CDK, cyclin-dependent kinase

**Fig. 3:**
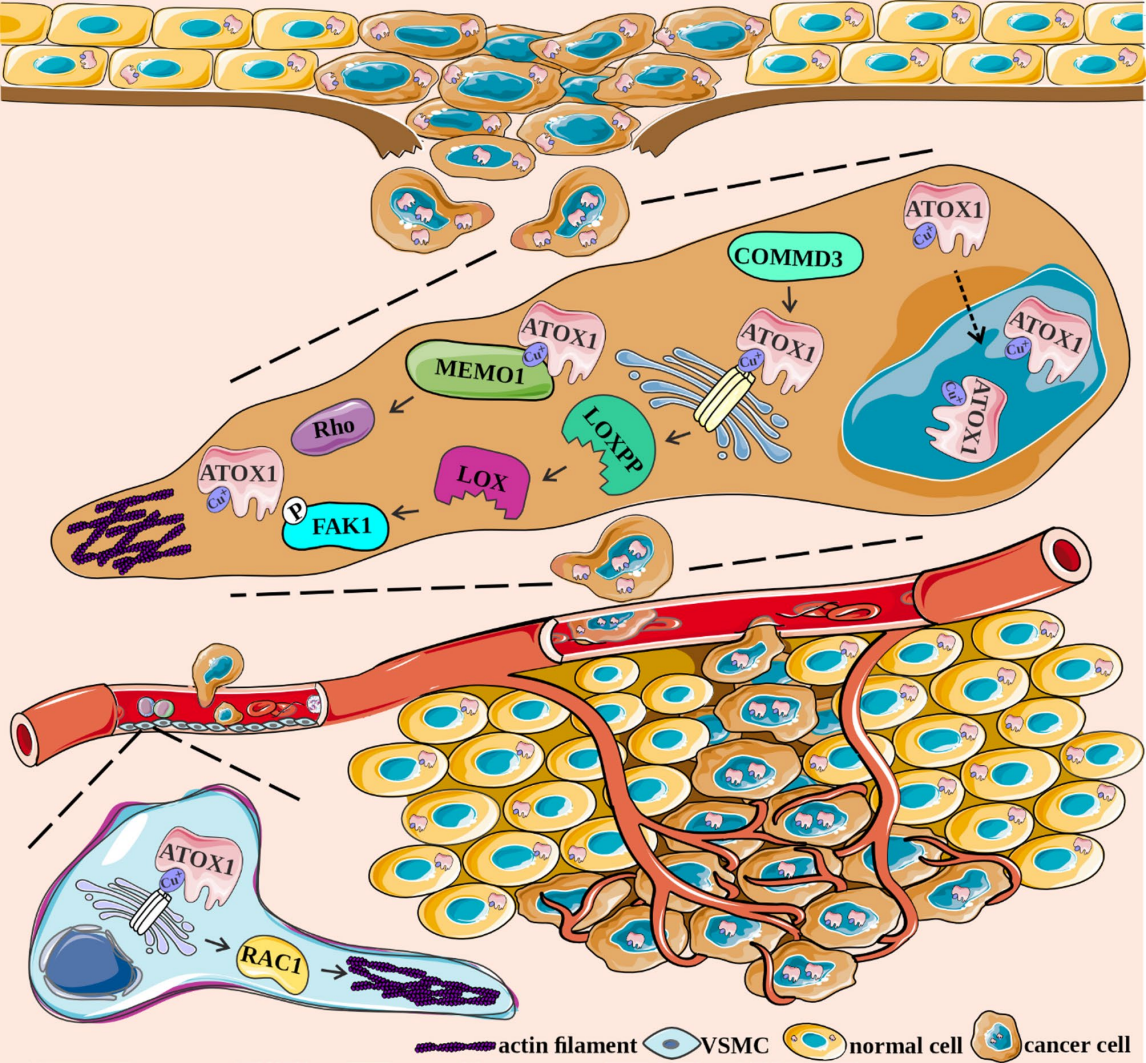
The metastasis-promoting activities of ATOX1. In metastatic cancer cells, ATOX1 is predominantly localized in the nucleus. In addition, ATOX1 accumulates at the lamellipodial margins of migrating cancer cells. The ATOX1-ATP7A-LOX axis plays an important role in migrating cells and contributes to downstream pro-metastatic FAK/SRC signaling. COMMD3 may also be involved in the regulation of this axis. In addition, ATOX1 may be associated with microtubule guidance, actin network and adhesion site formation in migrating cells through interaction with MEMO1 and the MEMO-RhoA-mDia1 cascade. ATOX1 may also contribute to vascular remodeling and thus angiogenesis by interacting with the RAC1 protein in VSMC. COMMD3, COMM domain-containing protein 3; FAK1, focal adhesion kinase 1; LOXPP, a precursor of LOX; MEMO1, mediator of cell motility 1; RAC1, RAS-related C3 botulinum toxin substrate 1; RhoA, Ras homolog family member A; VSMC, vascular smooth muscle cells

**Fig. 4:**
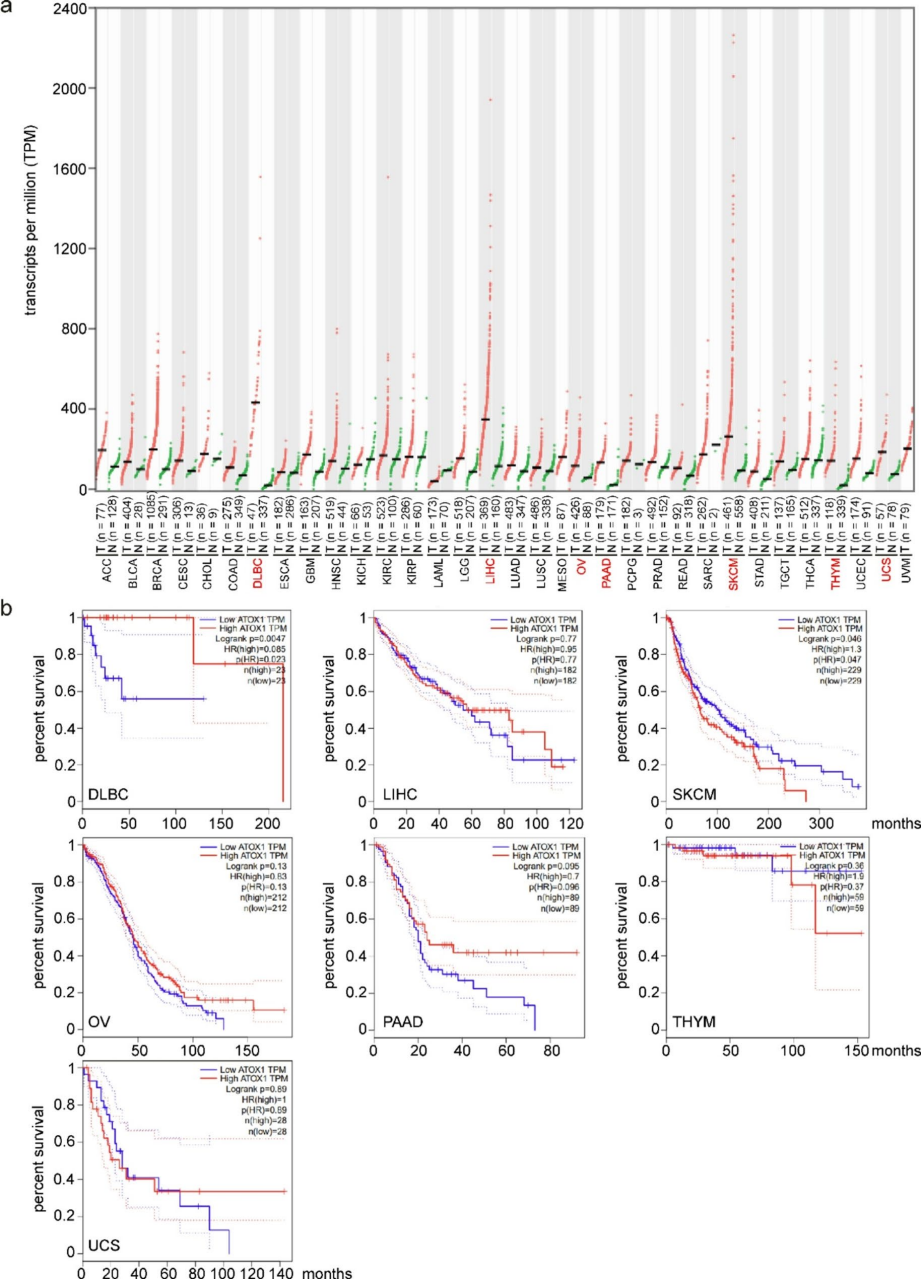
(**a**) Transcript levels of *ATOX1* were retrieved from Gene Expression Profiling Integrative Analysis (GEPIA) and shown for different types of cancer. Cancers with significantly higher *ATOX1* mRNA levels compared with normal cells are marked in red. n – number of samples (**b**) Overall survival of patients with cancers marked in red in panel (**a**). The median level of *ATOX1* mRNA was used as a group cutoff. ACC, adrenocortical carcinoma; BLCA, bladder urothelial carcinoma; BRCA, breast invasive carcinoma; CESC, cervical squamous cell carcinoma and endocervical adenocarcinoma; CHOL, cholangiocarcinoma; COAD, colon adenocarcinoma; DLBC, diffuse large B-cell lymphoma; ESCA, esophageal carcinoma; GBM, glioblastoma multiforme; HNSC, head and neck squamous cell carcinoma; KICH, kidney chromophobe; KIRC, kidney renal clear cell carcinoma; KIRP, kidney renal papillary cell carcinoma; LAML, acute myeloid leukemia; LGG, brain lower grade glioma; LIHC, liver hepatocellular carcinoma; LUAD, lung adenocarcinoma; LUSC, lung squamous cell carcinoma; MESO, mesothelioma; OV, ovarian serous cystadenocarcinoma; PAAD, pancreatic adenocarcinoma; PCPG, pheochromocytoma and paraganglioma; PRAD, prostate adenocarcinoma; READ, rectum adenocarcinoma; SARC, sarcoma; SKCM, skin cutaneous melanoma; STAD, stomach adenocarcinoma; TGCT, testicular germ cell tumors; THCA, thyroid carcinoma; THYM, thymoma; UCEC, uterine corpus endometrial carcinoma; UCS, uterine carcinosarcoma; UVM, uveal melanoma

#### Cellular localization of ATOX1

ATOX1 is predominantly localized in the cytoplasm due to its copper chaperone function, although ATOX1 has also been found in the nuclei of different types of cells owing to the presence of the NLS [34–37]. ATOX1 can also be shuttled to the nucleus in a tumor necrosis factor (TNF) receptor-associated factor 4 (TRAF4)-facilitated fashion as exemplified in endothelial cells as a result of the TNF-α-stimulated ROS-dependent inflammatory response [38]. A shift in the subcellular localization of ATOX1 to the nucleus can contribute to cancer spread [39]. This phenomenon can be, however, cancer type-specific as in melanoma cells, ATOX1 is predominantly localized in the cytosolic fraction [40]. Mutations in the metal-binding domain (MBD) of ATOX1 can block its nuclear translocation [39], highlighting the critical role of copper binding in the nuclear function of ATOX1. In line with these findings, copper-dependent homodimerization was previously demonstrated to be a key step in the regulation of ATOX1-dependent transcription [41]. In this context, conflicting observations on the function of ATOX1 in the nucleus have been reported. ATOX1 is a transcription factor that regulates the expression of several genes, including *CCND1* [32], superoxide dismutase 3 (*SOD3*) [34, 42, 43], neutrophil cytosolic factor 1 (*NCF1*) encoding p47^phox^, an NADPH oxidase organizer [44], a mediator of DNA damage checkpoint protein 1 (*MDC1*) [45], and inorganic pyrophosphatase 2 (*PPA2*) [46] (Table 1). A more recent study identified 248 genes involved in the inflammatory response and angiogenesis that are directly regulated by nuclear ATOX1 [36]. On the other hand, ATOX1 was detected in the nucleus of HeLa cells but no binding to DNA was assessed in vitro [35], suggesting that ATOX1 can indirectly affect the transcription of selected genes.

**Table 1:** Experimentally validated transcriptional target genes of ATOX1

Gene	Cell line	Method	Ref.
*CCND1*	mouse embryonic fibroblasts (MEFs)	ChIP assay EMSA	[32]
*MDC1*	pancreatic adenocarcinoma cells (Patu 8988T)	ChIP assay	[45]
*NCF1*	human umbilical vein endothelial cells (HUVECs)	ChIP assay	[44]
*PPA2*	sheep fetal fibroblasts	ChIP assay EMSA	[46]
*SOD3*	mouse embryonic fibroblasts (MEFs)	ChIP assay	[32]
	human leukemia monocytic cells (THP-1)	ChIP assay	[40]
	vascular smooth muscle cells (VSMCs)	DNA pull-down assay	[43]

EMSA, electrophoretic mobility shift assay; ChIP, chromatin immunoprecipitation assay

#### Functional partners of ATOX1 involved in canonical signaling pathways associated with intracellular copper homeostasis

ATOX1 interacts with several proteins predominantly through its role in the regulation of copper homeostasis and the management of oxidative stress, as revealed by STRING interactions (Fig. 1b). In this respect, CTR1 is a high-affinity copper uptake protein harboring two His-Met-Asp residues at the N-terminus that bind cuprous ions and maintain their reduced state [47]. A study on ATOX1 and the CTR1 carboxyl-terminal moiety has shown that Cu^+^ can be transferred directly from CTR1 to ATOX1 as evidenced by molecular docking and crystallography [29]. Furthermore, the interaction between ATOX1 and CCS has been shown to transfer copper ions from one protein to another [48]. In addition, glutaredoxin 1 and GSH can also supply copper ions for ATOX1 [49, 50]. Upon binding cuprous ions, ATOX1 is responsible for the trafficking of Cu^+^ to copper-transporting ATPases alpha (ATP7A) and beta (ATP7B), which shuttle cuprous ions within the lumen of the trans-Golgi network for the maturation of copper-dependent enzymes and can control the efflux of excess copper [4, 51–53]. ATOX1 exchanges Cu^+^ with the N-terminal fragment of ATP7A and ATP7B, which contain six MBDs, whereas each MBD contains an MXCXXC motif that is known to coordinate cuprous ions via two cysteine residues [54, 55]. ATOX1 can also act as an inhibitor of ATP7A/ATP7B as this secretion process can be reversed at low intracellular concentrations of copper ions. In this respect, the apo-form of ATOX1 can remove copper ions from the MBDs of ATP7A and ATP7B [11, 56]. Consequently, the ATOX1-ATP7A/ATP7B axis modulates both the exclusion of excess copper ions and the metalation of copper-dependent proteins [12, 57]. This is substantiated by the extensively discussed model demonstrating that the gradient of increasing copper-binding affinities of copper-binding proteins predominantly determines the directional trafficking of Cu^+^ to specific intracellular compartments. In this respect, distinct proteins involved in copper homeostasis exert various copper affinities, e.g., CTR1 (K_d_ >3 × 10^− 6^ M), GSH (K_d_ = 9 × 10^− 12^ M), and COX17 (K_d_ = 2 × 10^− 14^ M) [12]. As ATOX1 has been suggested to play a crucial role in decoding the cellular redox status to provide appropriate adjustment of intracellular copper homeostasis [12], these interactions emphasize the complex role of ATOX1 and the variability of proteins that are responsible for maintaining copper homeostasis.

### The role of ATOX1 in cancer

Since ATOX1 is involved in copper ion distribution within cells, the dysregulation of ATOX1-dependent processes may contribute to oxidative stress leading to inflammation and inflammation-related diseases such as inflammatory bowel disease and ulcerative colitis, neurodegenerative disorders and cancer [58–65]. In this respect, ATOX1 is also closely associated with several features of cancer development and progression, including uncontrolled cell proliferation, angiogenesis, stimulation of invasion and metastatic spread, disruption of cellular metabolic processes and drug resistance [33, 38, 66, 67]. Since many types of cancer are characterized by increased levels of intratumoral copper [68], ATOX1-dependent regulation of the phenotype of cancer cells can be largely associated with the protumorigenic role of copper known as cuproplasia [57, 69]. In this respect, owing to its metallochaperone and transcription-associated activities, the contribution of ATOX1 to multiple cancer-related processes is related to the regulation of the functions of several cancer cell-associated proteins.

#### ATOX1 and proliferation of cancer cells

Owing to its nuclear function, ATOX1 may directly regulate the expression of genes, including cell cycle-related genes such as *CCND1* encoding cyclin D1, which is a crucial mediator of cell cycle progression (Fig. 2) [70]. An electrophoretic mobility supershift assay revealed that ATOX1 aids in copper transport into the nucleus via its conserved MBD and NLS domains and that ATOX1 binds to the − 535 to -530 (5’-GAAAGA-3’) region of the *CCND1* promoter. In turn, silencing *ATOX1* expression led to significant suppression of cell proliferation associated with decreased cyclin D1 levels and arrested the G1-S phase transition in mouse embryonic fibroblasts [32]. Suppression of cell proliferation in response to RNAi-mediated knockdown of ATOX1 was also found in non-small cell lung cancer [71], melanoma [40] and colorectal cancer [39] cells. In turn, no significant decrease in cell proliferation was observed in normal human keratinocytes upon ATOX1 knockdown suggesting that ATOX1 can be particularly crucial for the proliferation of cancer cells [72]. In addition to the regulation of cyclin D1 levels, the contribution of ATOX1 to cell cycle progression can include its direct interaction with subunits of the anaphase-promoting complex (APC), as demonstrated in HEK293T and breast cancer cells (Fig. 2) [73]. A central regulatory function of ATOX1 in modulating cell cycle progression has been comprehensively reported in a recent study on diffuse large B-cell lymphoma (DLBCL) cell lines and primary DLBCL samples [74]. Depletion of ATOX1 markedly suppressed cell proliferation and led to cell cycle arrest at the G2 phase, whereas treatment with DC_AC50, a dual inhibitor of ATOX1 and CCS, elicited a dose-dependent antiproliferative response that was markedly enhanced by ATOX1 silencing [74]. Mechanistically, downregulation of ATOX1 led to a substantial reduction in the level of cyclin B1, a key regulator responsible for facilitating the transition from the G2 phase to the M phase of the cell cycle. Concomitantly, pronounced upregulation of p27^KIP1^, a cyclin-dependent kinase inhibitor that mediates G1 phase arrest, was observed [74, 75]. Recently, it has been shown that cyclin B1 contributes to the activation of cyclin-dependent kinase 1 (CDK1) by facilitating the transfer of copper ions from ATOX1 to CDK1 [76].

In addition to directly regulating the cell cycle machinery, ATOX1 influences other signaling proteins that are crucial for the regulation of cell proliferation (Fig. 2). The interaction between ATOX1 and p53 has been shown [77], confirming the role of p53 in copper transport into the nucleus [78]. Studies on colorectal cancer and lung carcinoma cells exposed to genotoxic stress revealed reduced ATOX1 levels in cells with functional p53, whereas p53-knockout cells presented significantly increased ATOX1 mRNA and protein levels [79]. In the case of the p53 inactivation, ATOX1 contributes to cell cycle progression via the upregulation of cyclin D1 in cells with DNA damage, whereas the downregulation of both p53 and ATOX1 results in cell death via apoptosis [79, 80]. Another example of an ATOX1-regulated signaling pathway involved in the control of excessive proliferation of cancer cells is the RAS-RAF-MEK-ERK cascade as mitogen-activated protein kinase kinase (MEK) has been identified as a copper-dependent kinase, whereas copper is an allosteric regulator of the interaction between MEK and extracellular signal-regulated kinase 1/2 (ERK1/2) [81, 82]. Notably, genetic alterations result in the abnormal activity of the RAS-RAF-MEK-ERK pathway in several types of cancers [83, 84]. In this respect, ATOX1 is critical for the activation of the MEK-ERK signaling pathway in BRAF^V600E^-mutated melanomas, as the pharmacological inhibition of ATOX1 by DC_AC50 decreases the phosphorylation of ERK1/2 and reduces the growth of *BRAF*-mutated melanoma cells [40]. CRISPR/Cas9-mediated *ATOX1* editing was accompanied by decreased ERK1/2 phosphorylation without disturbing the activity of other MAPK proteins such as c-Jun N-terminal kinase (JNK) and p38 [40]. Accordingly, a similar effect of ATOX1 downregulation has been recently reported in DLBCL cells, whereas copper supplementation in *ATOX*-knockout cells was sufficient to restore ERK1/2 activity [74].

#### ATOX1 and cancer cell migration, invasion and angiogenesis

Metastasis is a multistep process that primarily accounts for death in patients with advanced cancer [85, 86]. Several copper-dependent proteins have been associated with the metastatic spread of cancer cells, and high levels of ATOX1 in cancer cells can promote their migratory and invasive properties [87–89]. ATOX1 can also be involved in metastasis-supporting events in noncancer cells [37, 44], for example by contributing to the recruitment of inflammatory cells as it functions as a transcriptional regulator of *NCF1* encoding p47^phox^ [44]. The nuclear function of ATOX1 has also been investigated in a study comparing colorectal cancer cells with metastatic potential (SW620) and nonmetastatic cells (SW480), and increased accumulation of ATOX1 protein in the nuclear fractions of SW620 cells has been reported [39]. In addition, treatment with activin A, a member of the transforming growth factor beta (TGF-β) family involved in cancer cell migration and epithelial-to-mesenchymal transition, promoted the nuclear translocation of ATOX1 in both nonmetastatic and metastatic cell lines. Conversely, ATOX1 knockdown in SW620 cells resulted in decreased cell migration and reduced colony formation, whereas upregulating ATOX1 levels in SW420 cells significantly increased their migration and colony-forming capacity [39]. ATOX1 supports the metalation of copper-dependent enzymes involved in several signaling pathways that control migration- and invasion-related activities, such as cell adhesion, cytoskeletal remodeling and extracellular matrix (ECM) degradation (Fig. 3) [90, 91]. As local cell movement is facilitated by the formation of actin filament-enriched extensions of the plasma membrane at the leading edge of the cell as exemplified by lamellipodia [92], ATOX1 accumulates at lamellipodia borders of migrating breast cancer cells [87]. In this respect, although downregulation of ATOX1 inhibited cell migration as evidenced by reduced wound closure in a scratch assay, the formation of lamellipodia was not affected [87]. It has also been demonstrated that ATOX1 may deliver copper ions to lysyl oxidase (LOX) via ATP7A at the lamellipodia border [93]. As the copper-dependent LOX family of proteins plays a crucial role in catalyzing the cross-linking of collagen and elastin, which enhances cell migration and facilitates tumor progression [91, 94–96], the ATOX1-ATP7A-LOX cascade is expected to contribute to ECM remodeling in the tumor microenvironment. Accordingly, immunofluorescence staining revealed partial colocalization of ATOX1, ATP7A and LOXPP, c, at the lamellipodial borders of breast cancer cells [88]. The assessment of protein-protein interactions via in situ proximity ligation assays revealed a significant reduction in the number of ATOX1-LOXPP interactions following the downregulation of ATP7A, whereas a similar decrease in the ATP7A-LOXPP interaction was detected upon the silencing of ATOX1 [88]. It has also been shown in mouse mammary carcinoma cells that downregulation of *ATP7A* led to the loss of LOX-mediated phosphorylation of focal adhesion kinase 1 (FAK1), a key regulator of tumor cell adhesion and motility [97]. Further research is necessary to delineate the role of ATOX1 in cancer cell migration, as the ATOX1-ATP7A-LOX axis and copper metabolism pathways were recently identified as COMM domain-containing protein 3 (COMMD3)-regulated signaling cascades involved in the progression of multiple myeloma [98]. In contrast, COMMD3 was previously identified as a negative regulator of the invasive phenotype of breast cancer cells [99]. While the role of ATOX1 has not been investigated in this context [99], the cancer type-specific contribution of ATOX1-related partners to cancer cell migratory and invasive potential points to a presumably complex role of ATOX1 in these processes. These findings are substantiated by studies showing that ATOX1 can also contribute to other processes that support the efficient metastasis of cancer cells. In this respect, ATOX1 plays an important role in vascular remodeling by promoting copper- and RAS-related C3 botulinum toxin substrate 1 (RAC1)-dependent migration of vascular smooth muscle cells, macrophage infiltration and LOX activation [93], thereby contributing to tumor angiogenesis. Accordingly, ATOX1 inhibition prevents angiogenesis and tumor growth in a xenograft mouse model of breast cancer [100]. It has also been shown that ATOX1 can interact with mediator of cell motility 1 (MEMO1), which coordinates microtubule dynamics, actin cytoskeletal remodeling, and adhesion complex formation, all of which are essential for cell migration [101, 102]. In this respect, MEMO1 is required for breast carcinogenesis [101] as well as the migration and invasion of breast cancer cells and their metastasis in vivo [102, 103]. Recently, MEMO1 was shown to contribute to the regulation of intracellular copper homeostasis by binding to Cu(I)-loaded ATOX1 (Kd = 2.1 × 10^− 7^ M), as demonstrated in real-time by surface plasmon resonance, whereas the transfer of cuprous ions from Cu(I)-loaded ATOX1 to MEMO1 has been confirmed to prevent copper-dependent ROS-mediated cytotoxicity. In breast cancer cells, the functional proximity of ATOX1 and MEMO1 was demonstrated although no interaction was observed between apo-ATOX1 and MEMO1 [104].

#### ATOX1 and oxidative stress

As ATOX1 is a crucial intracellular copper chaperone, ATOX1 inhibition in cancer cells increases total cellular copper and ROS levels, which is associated with decreased SOD1 activity and ATP synthesis as well as lower NADPH and GSH levels, leading to impaired ROS clearance [72]. In addition, cyclooxygenase 1 (COX1) and COX2 activities and oxygen consumption are downregulated [72]. It has also been reported that ATOX1 transcriptionally regulates *SOD3* expression in diverse types of cells, including tumor-associated macrophages [34, 42, 105], suggesting that ATOX1 may play a role in tumor progression [106, 107]. Mechanistically, a shift in the intracellular distribution of ATOX1 to the nucleus has been demonstrated in response to 12-O-tetradecanoylphorbol-13-acetate, and this translocation was reversed by copper chelators, MEK/ERK inhibitors and NADPH oxidase 2 (NOX2) inhibitors [42]. DC_AC50-dependent inhibition of ATOX1 and reduced GSH levels increase the vulnerability of basal-like breast cancer cells to copper imbalance and oxidative stress [100]. A recent study in normal cells revealed that inactivation of Hippo-YAP signaling promoted cell survival via the upregulation of ATOX1, which was followed by increased copper efflux and increased antioxidant capacity. Mechanistically, ATOX1 acts as a transcription factor that regulates the expression of the mitochondrial gene *PPA2*, which is involved in the scavenging of excess ROS [46]. It remains, however, to be verified in cancer cells regarding the crucial role of the Hippo-YAP signaling in these cells [108–110]. Another mechanism linking oxidative stress and ATOX1 in cancer cells has been shown using the proximity-tagging of ATOX1 by ascorbate peroxidase 2. Cysteine-rich secretory protein 2 (CRIP2) has been identified as a nuclear interacting partner for ATOX1 in lung cancer cells. In this respect, ATOX1 transfers copper ions to CRIP2 leading to changes in the secondary structure of CRIP2 followed by its degradation. Consequently, increases in ROS levels and autophagy have been reported [111]. However, it remains to be determined whether ATOX1 and CRIP2 bind copper competitively [112]. As autophagy can have a dual role in cancer cells by either a protumorigenic or a cell death-inducing process [113–120], the further role of ATOX1 in this respect may be crucial from a therapeutic point of view. This is additionally supported by the fact that Unc-51 like autophagy activating kinases 1/2 (ULK1/2), basic components of the autophagic machinery, are copper-dependent proteins [121], and a complex interplay between copper metabolism and autophagy has been recognized [122].

#### ATOX1 and the response to therapy and drug resistance of cancer cells

The clinical efficacy of standard chemotherapy is limited by the resistance of cancer cells due to a number of mechanisms [123]. Copper homeostasis is critical for the efficacy of platinum-based chemotherapy, and ATOX1 may play a key role in the development of drug resistance in cancer cells [124]. In this respect, elevated expression of *ATOX1* has been linked to increased tolerance to cisplatin in several types of cancer cell lines, including melanoma [45, 125, 126]. Additionally, ATOX1 levels are significantly higher in cisplatin-resistant melanoma cells than in cisplatin-sensitive ovarian carcinoma cells [126]. A two-hour exposure to cisplatin also leads to an upregulation of ATOX1 [126]. Mechanistically, cisplatin can enter cells via CTR1 and be transferred to ATOX1 in the cytoplasm following a conformational change in the ATOX1-drug complex that induces its translocation to ATP7A and ATP7B for efflux [126–130]. This can be considered a possible mechanism of drug detoxification by cancer cells. Copper binding significantly enhances the interaction between ATOX1 and cisplatin [131] via forming the ATOX1-Cu-Pt tertiary complex through sulfur-bridge linkages [132]. Cisplatin binds to oxidized Cu(I)-ATOX1 by targeting a methionine residue of ATOX1. In the presence of excess GSH, cisplatin binds irreversibly to ATOX1 via Cys12 and Cys15 [133]. Ligands around the platinum-center in platinum-based drugs have essential roles in tuning interactions with ATOX1 [134]. In turn, tetrathiomolybdate (TTM), a copper chelator, can prevent cisplatin-induced protein unfolding and aggregation by inhibiting the interaction between cisplatin and ATOX1 [135, 136]. Furthermore, the knockdown of ATOX1, but not CCS, significantly increased the sensitivity of pancreatic adenocarcinoma cells to the genotoxic drugs gemcitabine and adriamycin [45]. Accordingly, substantial cancer cell death and inhibition of tumor growth were demonstrated following treatment with DC_AC50 and gemcitabine [45]. Notably, these cancer cells presented increased levels of ATOX1 after pressure selection with a high dose of gemcitabine, while ATOX1 knockdown in drug-resistant cells sensitized them to gemcitabine [45]. Mechanistically, ATOX1 binds to the 5’-TGAAA-3’ region of the *MDC1* promoter and activates its transcription in a copper-dependent manner [45]. MDC1 promotes double-strand DNA repair [137–139] and regulates transcription as a regulator of RNA polymerase II [140]. Accordingly, a positive correlation between the levels of ATOX1 and MDC1 in pancreatic tumor tissues was detected via the Gene Expression Profiling Interactive Analysis (GEPIA) tool [45, 141]. An increased efficacy of carboplatin [142] and paclitaxel [100] was also found in osteosarcoma or triple-negative breast cancer cells after ATOX1 inhibition by DC_AC50. The contribution of ATOX1 to the regulation of ovarian cancer cell sensitivity to cisplatin has been questioned by others using CRISPR-Cas9-mediated knockout of *ATOX1* [143]. In contrast to the relatively well-established role of ATOX1 in the response of cancer cells to standard chemotherapy, the contribution of ATOX1 to cell susceptibility to other classes of anticancer therapeutics remains elusive. For example, the siRNA-mediated knockdown of ATOX1 in wild-type *BRAF* melanoma cells significantly diminished the accumulation of copper ions and reduced the susceptibility of cells to combination therapy with trametinib and disulfiram [144]. As trametinib is a MEK1/2 inhibitor used in the clinic and has also been assessed in preclinical studies as a part of drug combinations [145, 146], and disulfiram is an FDA-approved drug for alcohol addiction that has shown potential anticancer activity as a copper ionophore [147–149], this indicates that ATOX1 can be an important protein potentially determining the efficacy of copper-related therapeutic approaches. In this respect, potential targeting ATOX1 to improve the anticancer efficacy of other drugs or drug combinations might be feasible via the use of DC_AC50 and other, more selective ATOX1-targeted strategies. The outcomes of ATOX1 level modulation with regard to the response of cancer cells to distinct drugs are summarized in Table 2.

**Table 2:** Molecular and cellular effects in cancer cells following the modulation of ATOX1 levels

Effect on ATOX1 level	Affecting factor	Cancer type	Molecular / cellular effects	Ref.
downregulation	DC_AC50, shRNA, CRISPR knockout	pancreatic cancer	increased sensitivity to gemcitabine and adriamycin inhibition of tumor growth	[45]
downregulation	DC_AC50	triple-negative breast cancer	inhibition of tumor growth, suppression of angiogenesis in a xenograft mouse model, diminished formation of endothelial cell network, potentiation of paclitaxel cytotoxicity	[100]
downregulation	DC_AC50	osteosarcoma	cell cycle arrest, potentiation of carboplatin-induced apoptosis, reduced migratory potential of cells	[142]
downregulation	siRNA	melanoma	reduced intracellular accumulation of copper ions, reduced cell susceptibility to a combination of trametinib and disulfiram	[144]
upregulation	cisplatin	melanoma, ovarian cancer	increased resistance of cells to cisplatin	[126]

### ATOX1 as a potential biomarker in cancer

Considering the extensive role of ATOX1 in distinct key functions of cancer cells, the ATOX1 transcript and/or protein levels might be considered to have prognostic value. *ATOX1* is overexpressed in breast cancer [90, 100], colon cancer [39], lung cancer [71, 79], melanoma [40], and multiple myeloma [98]. The overexpression of *ATOX1* has been correlated with poor patient survival [40, 98], while cellular localization of ATOX1 can be crucial for cancer cell fate [39]. In turn, a high level of cytoplasmic ATOX1 can be considered a predictive biomarker for TTM treatment in breast cancer patients at high risk of recurrence [89], although high levels of *ATOX1* mRNA are associated with a worse prognosis for breast cancer patients when early stages of disease and estrogen receptor (ER)-positive subtypes are considered [150]. *ATOX1* is a cuproptosis-related gene that is overexpressed in lung adenocarcinoma (LUAD) compared with normal tissue [151]. *ATOX1* has been identified as one of six genes involved in the progression of DLBCL, and it is the most promising gene for disease diagnosis [74]. To substantiate the potential role of ATOX1 in cancer, we retrieved expression data from GEPIA for a broad panel of cancer types and compared the expression of *ATOX1* in cancer cells with its level in normal tissues. This analysis confirmed that *ATOX1* mRNA levels are generally increased in the cancers with the most significant upregulation in DLBC, liver hepatocellular carcinoma, ovarian serous cystadenocarcinoma, skin cutaneous melanoma, pancreatic adenocarcinoma, thymoma, and uterine carcinosarcoma (Fig. 4a). Surprisingly, when Kaplan-Meier survival analysis was performed for patients with these types of cancer, overall survival was significantly greater in both ATOX1^high^ DLBC patients and ATOX1^low^ skin melanoma patients (Fig. 4b).

## Conclusions and future perspectives

Research on ATOX1 has demonstrated its multifaceted roles and the complexity of copper ion homeostasis in cells, but it has also revealed how copper can influence diseases, including cancer, at the molecular level [152]. As copper homeostasis is frequently affected in cancer patients, ATOX1 can contribute to the adaptation of cancer cells to dysregulated copper levels [153]. In this respect, further research is necessary to more comprehensively and systematically delineate the molecular and cellular causes and corresponding effects of both the cytosolic action and nuclear redistribution of ATOX1 as well as its transcriptional targets. How the transcriptional activity of ATOX1 is regulated and what kinds of stimuli, in addition to copper ions, shift their intracellular distribution from the cytosol to the nucleus remain open questions. In addition, recently identified genes regulated by nuclear ATOX1 [36] should be more comprehensively validated with respect to their direct transcriptional dependence on ATOX1. Additionally, the role of ATOX1 in other cellular processes such as chaperone-mediated autophagy [154–156] may enable the identification of novel functions of ATOX1.

Modulating copper availability has shown anticancer activity, and different compounds affecting copper levels in cancer are currently being tested in clinical trials (Table 3). In this respect, copper chelation [157, 158] and excessive copper levels achieved by the use of copper ionophores [53, 159] were previously associated with cancer cell death, including cells exhibiting resistance to drugs currently used in the clinic. Importantly, ATOX1 has been shown to play a central role in cell sensitivity to copper chelators and ionophores [160]. For this reason, investigations of the anticancer activity of copper ionophores such as elesclomol [161] and the repurposed use of disulfiram [147–149] should also focus on assessing the role of ATOX1 and determining the particular type of cell death induced, including a novel type of regulated cell death, cuproptosis [162–166]. Recent studies showing that (a) the overexpression of lipoyltransferase 1 (LIPT1), a gene associated with cuproptosis, downregulates ATOX1, thereby impeding the progression of lung cancer [167], and (b) ATOX1 can interact with curcumin [168] emphasize that further studies on other interactions and binding partners for ATOX1 are needed. In this respect, ATOX1-interacting proteins that have been identified in a yeast two-hybrid screen with a large human placenta library of cDNA fragments [169] should be further validated. These interactions may be crucial for the development of efficient therapeutic approaches using combinations of distinct classes of anticancer drugs. Although the contribution of ATOX1 to the cancer cell sensitivity to standard chemotherapy has been recognized, the role of ATOX1 in the response and resistance of cancer cells to modern targeted therapies and immunotherapy remains to be elucidated. This is expected and substantiated by the role of ATOX1 in regulating the redox status of cancer cells and their response to therapy [170]. Moreover, an increasing number of studies have revealed (a) the role of copper-dependent pathways in immune evasion by cancer cells [171, 172], (b) copper-dependent proteins as targets to combat resistance to immunotherapy as exemplified by ULK1, which contributes to interferon-gamma-induced immunosuppressive genes [173, 174], (c) the significant role of ATOX1 in cancer inflammation by modulating immune responses as ATOX1 can promote M1 polarization of macrophages [62] and influence the migration of immune cells such as macrophages and neutrophils to tumor sites by regulating key signaling pathways such as nuclear factor-kappa B (NF-κB) and p38, ERK and c-JUN [175], and (d) the restoration of the immunogenicity of tumor cells in response to ATOX1 inhibition [176]. Therefore, novel therapeutic strategies involving copper [177] and ATOX1 modulation [178] might be largely considered in the treatment of cancer patients.

**Table 3:** The current clinical trials for copper ionophores and copper chelators in cancer patients

Agents	Role	Combination	Condition	Status	Phase	ID
Disulfiram (DSF)	copper ionophore	copper suplement	breast neoplasm female metastatic breast cancer	recruiting	phase 2	NCT03323346
		cisplatin	advanced gastric cancer	not yet recruiting	not applicable	NCT05667415
		copper gluconate, liposomal and doxorubicin (Doxil)	relapsed sarcomas	recruiting	phase 1	NCT05210374
Tetrathiomolybdate (TTM)	copper chelator	capecitabine pembrolizumab	triple-negative breast cancer	recruiting	phase 1 phase 2	NCT06134375
Penicillamine	copper chelator	-	head and neck cancer	recruiting	phase 2	NCT06103617

accessed 12th July 2025

## Data Availability

No datasets were generated or analysed during the current study.

## Abbreviations

APC: Anaphase-promoting complex
ATOX1: Antioxidant protein 1
ATP7A: Copper-transporting ATPase alpha
ATP7B: Copper-transporting ATPase beta
CCND1: Cyclin D1
CCS: Copper chaperone for superoxide dismutase
CDK1: Cyclin-dependent kinase 1
COMMD3: COMM domain-containing protein 3
COX: Cyclooxygenase
CRIP2: Cysteine-rich secretory protein 2
CTR1: Copper transporter 1
CuL: Copper-complexing ligand
DLBCL: Diffuse large B-cell lymphoma
ECM: Extracellular matrix
ER: Estrogen receptor
ERK1/2: Extracellular signal-regulated kinase 1/2
FAK1: Focal adhesion kinase 1
GEPIA: Gene expression profiling interactive analysis
GSH: Glutathione
JNK: c-Jun N-terminal kinase
LIPT1: Lipoyltransferase 1
LOX: Lysyl oxidase
LUAD: Lung adenocarcinoma
MDC1: Mediator of DNA damage checkpoint protein 1
MEK: Mitogen-activated protein kinase kinase
MEMO1: Mediator of cell motility 1
NCF1: Neutrophil cytosolic factor 1
NF: κB-nuclear factor-kappa B
NLS: Nuclear localization sequence
NOX2: NADPH oxidase 2
PPA2: Inorganic pyrophosphatase 2
RAC1: RAS-related C3 botulinum toxin substrate 1
ROS: Reactive oxygen species
SOD3: Superoxide dismutase 3
TGF-β: Transforming growth factor beta
TNF: Tumor necrosis factor
TRAF4: TNF receptor-associated factor 4
TTM: Tetrathiomolybdate
ULK1/2: Unc-51 like autophagy activating kinases 1/2
